# Common predictors of spoken and written language performance in aphasia, alexia, and agraphia

**DOI:** 10.3389/fnhum.2022.1025468

**Published:** 2022-11-07

**Authors:** Pélagie M. Beeson, Kindle Rising, Alyssa Sachs, Steven Z. Rapcsak

**Affiliations:** ^1^Department of Speech, Language, and Hearing Sciences, The University of Arizona, Tucson, AZ, United States; ^2^Department of Neurology, The University of Arizona, Tucson, AZ, United States; ^3^Banner Alzheimer’s Institute, Tucson, AZ, United States

**Keywords:** aphasia, phonological agraphia, phonological alexia, writing, spelling, dorsal language pathway, naming, reading

## Abstract

Language performance requires support from central cognitive/linguistic abilities as well as the more peripheral sensorimotor skills to plan and implement spoken and written communication. Both output modalities are vulnerable to impairment following damage to the language-dominant hemisphere, but much of the research to date has focused exclusively on spoken language. In this study we aimed to examine an integrated model of language processing that includes the common cognitive processes that support spoken and written language, as well as modality-specific skills. To do so, we evaluated spoken and written language performance from 87 individuals with acquired language impairment resulting from damage to left perisylvian cortical regions that collectively constitute the dorsal language pathway. Comprehensive behavioral assessment served to characterize the status of central and peripheral components of language processing in relation to neurotypical controls (*n* = 38). Performance data entered into principal components analyses (with or without control scores) consistently yielded a strong five-factor solution. In line with a primary systems framework, three central cognitive factors emerged: semantics, phonology, and orthography that were distinguished from peripheral processes supporting speech production and allographic skill for handwriting. The central phonology construct reflected performance on phonological awareness and manipulation tasks and showed the greatest deficit of all the derived factors. Importantly, this phonological construct was orthogonal to the speech production factor that reflected repetition of words/non-words. When entered into regression analyses, semantics and phonological skill were common predictors of language performance across spoken and written modalities. The speech production factor was also a strong, distinct predictor of spoken naming and oral reading, in contrast to allographic skills which only predicted written output. As expected, visual orthographic processing contributed more to written than spoken language tasks and reading/spelling performance was strongly reliant on phonological and semantic abilities. Despite the heterogeneity of this cohort regarding aphasia type and severity, the marked impairment of phonological skill was a unifying feature. These findings prompt greater attention to clinical assessment and potential treatment of underlying phonological skill in individuals with left perisylvian damage.

## Introduction

It has been nearly 25 years since the primary systems hypothesis was put forth positing that performance on spoken and written language tasks is the reflection of interactive processing among a limited number of cognitive systems (Joanisse and Seidenberg, 1999; Patterson and Lambon Ralph, 1999). Initially framed as an explanatory model of reading and acquired alexia, the primary systems approach characterized reading impairment as the disruption of one or more cognitive components that are not exclusive to reading. Specifically, the triangle/connectionist model of reading postulated that reading ability is dependent on the status of conceptual knowledge (semantics), the sound system (phonology), and vision (Plaut et al., 1996; Patterson and Lambon Ralph, 1999). Since this initial work, the primary systems framework has been extended to account for an array of behaviors documented in individuals with acquired language impairment as well as a means to examine neural support for common language components. Studies have consistently demonstrated that performance on spoken language tasks reflects the status of central semantic and phonological processing components (Lambon Ralph et al., 2002; Kümmerer et al., 2013; Butler et al., 2014; Mirman et al., 2015a,b; Halai et al., 2017; Tochadse et al., 2018). The written modality of language output has received limited attention in relation to building and testing primary systems models (for exceptions, see Henry et al., 2007, 2012; Rapcsak et al., 2009). Given that literate adults engage both spoken and written language in everyday life, a primary systems framework for language processing should reflect both modalities. In addition, a more complete model of language processing should also address the contribution of peripheral sensorimotor processes that support speech production and written communication.

The reality that central language impairment affects both spoken and written modalities is clearly evident in individuals with aphasia following left middle cerebral artery stroke. This most common cause of aphasia results from damage to critical left perisylvian regions and considerable attention has been directed toward the consequent impairment of speech production and auditory comprehension that characterize classic aphasia profiles (Kertesz, 1982; Goodglass and Kaplan, 1983; Benson and Ardila, 1996). Damage within this region also results in consistent patterns of acquired alexia and agraphia; specifically, phonological alexia and agraphia are prevalent following left perisylvian damage (Patterson and Marcel, 1992; Fiez et al., 2006; Henry et al., 2007; Rapcsak et al., 2009; Madden et al., 2018). The hallmark characteristic of these written language syndromes is a lexicality effect wherein real words are read or spelled better than non-words, with the latter requiring transcoding from letters to sounds and vice versa. Rapcsak et al. (2009) demonstrated that the underlying deficit is a common phonological impairment that is not specific to reading or spelling but is evident on tasks that require phonological awareness and manipulation skills apart from orthographic knowledge. In fact, the degree of phonological impairment as indexed by a composite score derived from a battery of relevant tasks was predictive of the severity of the acquired alexia and agraphia. In addition, a lexicality effect was evident on spoken repetition of words versus non-words, consistent with the notion of a central phonological deficit underlying both spoken and written language dysfunction. Taken together, these findings provide strong support for the primary systems hypothesis by demonstrating that phonological impairment has similar consequences for both spoken and written language performance.

The central phonological deficit that is prominent following left perisylvian damage stands in contrast to the semantic impairment that is well documented in the semantic variant of primary progressive aphasia (Lambon Ralph et al., 2001; Hodges and Patterson, 2007). Early investigations of a primary systems model of language processing demonstrated that degraded semantic knowledge modulates spoken naming and oral reading ability in semantic dementia (Plaut et al., 1996; Lambon Ralph et al., 2001). Because the cortical atrophy in the semantic variant of PPA largely affects anterior temporal lobe regions that are outside the left perisylvian zone, central phonological skills are relatively preserved so that reading and spelling are often accomplished with reliance on a phonologically based “sounding out” strategy. The resulting profiles of surface alexia and surface agraphia are characterized by disproportionate difficulty with irregularly spelled words which lead to phonologically plausible errors, such as reading *blood* as “blewed” or spelling “circuit” as *serkit*. These results also support the primary systems hypothesis by demonstrating that the status of semantic representations affects both spoken and written language performance.

The contrasting written language profiles of phonological alexia/agraphia versus surface alexia/agraphia reflect modulation of central phonological and semantic components of the language system that depend on distinct neural networks (Rapcsak et al., 2009; Henry et al., 2012, 2016; Rapcsak and Beeson, 2015). It is well established that a dorsal pathway supports phonology, speech production, as well as phonological short-term memory and phonological awareness (Hickok and Poeppel, 2007; Saur et al., 2008). The complementary ventral pathway that supports semantic processing encompasses left middle/inferior temporal cortex and temporal pole. The semantic network is broadly distributed, with contributions from left anterior inferior frontal gyrus (pars orbitalis) and angular gyrus that are within the left middle cerebral artery distribution, and some right hemisphere regions (Binder et al., 2009). These two pathways interact with a specialized region within ventral occipito-temporal cortex known as the visual word form area (VWFA) that supports orthographic processing during reading and spelling (Beeson P. et al., 2003; Rapcsak and Beeson, 2004; Dehaene and Cohen, 2011; Rapp et al., 2016).

Performance on tasks such as spoken naming, oral reading, and writing reflect the status of these central components moderated by peripheral sensorimotor skills. For spoken language this includes processing of auditory or visual input that prompts a response and the motor planning/implementation for speech production that are reliant on regions within the dorsal pathway that also supports central phonological skills. During reading, visual input is initially processed by posterior occipital areas and the information is transmitted to the VWFA. Written language output depends on the planning and implementation of hand movements for writing (or keyboarding or text messaging). Motor control for the hand depends on a distributed network that includes left intraparietal sulcus, premotor regions for the hand (Exner’s area), and hand area of the motor cortex (Beeson P. et al., 2003; Purcell et al., 2011; Rapcsak and Beeson, 2015). Thus, the central and peripheral aspects of spoken language are highly dependent on left perisylvian regions, while the peripheral aspects of written language processing are supported by regions outside the left perisylvian language/speech areas.

While considerable progress has been made over the past few decades toward the refinement of explanatory models of language, these models have been skewed toward the processing and production of spoken language to the exclusion of written language, especially spelling. The purpose of the present study was to explore the underlying cognitive processes and sensorimotor skills that support spoken as well as written language abilities. To do so, we employed the exploratory principal components analysis (PCA) method to extract the constructs that underlie performance on a range of language tasks. This data-driven approach, which has broad application to the study of large, multidimensional data sets (Jolliffe and Cadima, 2016), has been implemented with good success by a number of language researchers in recent years. PCA provides a means to reduce the information available from large neuropsychological test batteries that inherently reflect correlated measures to a set of derived uncorrelated variables that maximize the explained variance. The multistep process involves analysis of the variance distributed across all measures, followed by extraction of a limited set of components (factors) that explain a sufficient amount of the total variance. Varimax rotation is commonly used to ensure that factor scores are orthogonal to (or independent of) one another. In the context of language research, factor scores can be viewed as empirically derived measures of underlying cognitive processes that support language performance. For example, applying PCA to data from 10 tests collected from 21 individuals with post-stroke aphasia, Lambon Ralph et al. (2002) demonstrated that “anomia is simply a reflection of semantic and phonological impairments,” which appeared as the title of their paper.

Indeed, semantic and phonological factors have consistently emerged from principal component analyses of data from individuals with stroke-related language impairment. There is a lack of clarity, however, regarding the nature of the underlying phonological skills necessary to support language. Models that focus on spoken language have often failed to distinguish between central phonological knowledge/skill and the more peripheral processes that support speech production, nor do they capture peripheral skills that support written language. This is likely a reflection of the test batteries that provide the data for analysis. PCA requires the collection of consistent datasets across an adequate number of individuals in relation to the number of variables sampled. In most contexts, researchers collect data over an extended period of time to aggregate an adequate sample size, so they may be limited to the behavioral measures employed at the outset. Given that emergent factors reflect the specific dataset, it is useful to review the range of tasks entered into PCA as well as how they load on particular factors. This is relevant to the refinement of language models and the study of neural substrates of language processing that use derived factors from PCA in conjunction with voxel-based lesion correlation maps (e.g., Mirman et al., 2015a,b; Woollams et al., 2018; Dickens et al., 2019; Ingram et al., 2020).

A semantic processing factor consistently emerges from PCA of language tasks, with the most frequently reported tasks including spoken-word to picture matching (SWPM), written-word to picture matching (WWPM), and the conceptual matching task of the *Pyramids and Palm Trees* (PPT) picture version (Howard and Patterson, 1992) or the *Camels and Cactus Test* (CCT) (Bozeat et al., 2000). Although the picture matching tasks (PPT and CCT) might appear to be relatively “pure” tests of semantics because they do not require processing of spoken or written words, they also require a deductive or problem-solving component to determine the semantic relations depicted. This was evident in some studies that showed the CCT loading strongly on a non-verbal cognitive factor along with tasks that require visual problem-solving, *Ravens Coloured Progressive Matrices* (Raven, 1977) and the *Brixton Spatial Anticipation Test* (Burgess and Shallice, 1997; Butler et al., 2014; Halai et al., 2017; Tochadse et al., 2018). Synonym judgment tasks also load on the semantic factor, but not as strongly as the spoken- or written-word to picture matching tasks.

A “phonology” factor typically emerges as accounting for the greatest proportion of variance in PCA models of language processing. Spoken repetition of single words and non-words consistently load with the strongest weight on this factor, regardless of task variations such as immediate or delayed conditions (Kümmerer et al., 2013; Butler et al., 2014; Halai et al., 2017; Tochadse et al., 2018; Woollams et al., 2018). Repetition tasks that place a burden on phonological working memory (e.g., digit span tasks) also show strong loadings on phonology, although Tochadse et al. (2018) found that this task loaded on an auditory working memory factor that was distinct from repetition of single words or non-words. Among the phonological tasks used by prior investigators, only non-word reading requires segmental transcoding of letters to sounds (Lambon Ralph et al., 2002). Noticeably absent from the phonological tasks were those that require phonological awareness or manipulation skills such as those that children master as they learn to read and write (Chafouleas et al., 1997; Anthony and Francis, 2005; Hogan et al., 2005) and which are markedly impaired in phonological alexia and agraphia (Patterson and Marcel, 1992; Crisp and Lambon Ralph, 2006; Henry et al., 2007; Rapcsak et al., 2009; Beeson et al., 2010). Such tasks include, for example, the ability to blend individual sounds presented auditorily into a single-syllable word or non-word (as in /m/- /e/ – /b/ = “meb”), or the deletion of an individual sound from a consonant-vowel-consonant string (as in “say ‘bat,’ now take away the /b/”), or sound replacement (as in, “say, ‘bad,’ now change the /b/ to /d/”). Although such tasks seem a bit removed from everyday spoken and written language activities, they provide evidence of underlying phonological skill that is typically available to literate adults. In summary, most PCA models to date do not distinguish central processing of phonology from peripheral aspects of speech production that clearly influence performance on tasks with spoken output (but see Mirman et al., 2015a,b; Thye and Mirman, 2018). Furthermore, prior studies have not assessed phonological awareness skills critical for reading and spelling.

The purpose of the present study was to extend and potentially refine current language processing models to characterize the underlying cognitive processes and sensorimotor skills that support spoken and written language. To do so, we aggregated case series data reflecting comprehensive assessment from all the individuals with acquired language impairment due to left perisylvian damage who were evaluated in our lab over the past 16 years. Our goal was to derive an empirically based model of central and peripheral components of language processing, and to examine the prediction of single-word naming, oral reading, and written spelling using derived factor scores. Distinct from previous PCA studies, we included information regarding the status of phonological awareness and manipulation skills that are typically vulnerable to damage in left perisylvian regions. We expected the phonological and semantic factor scores to explain variance in spoken and written language tasks because the status of these central domains is relevant to both. By contrast, we hypothesized that peripheral predictors will differentially affect spoken versus written language performance.

## Materials and methods

### Participants

This case series analysis included data from comprehensive language evaluations from eighty-seven individuals with acquired language impairment due to brain damage. These data included some cases that were reported in previous research [13 from Henry et al. (2007) who were among the 31 from Rapcsak et al. (2009); 2 from Beeson et al. (2010); 4 from Beeson et al. (2018), and 1 from Beeson et al. (2019)]. Data from 38 neurotypical adults provided the basis for comparison. All participants gave informed consent to take part in a multisession language assessment using protocols approved by the Human Subjects Protection Program at The University of Arizona. The individuals with left hemisphere damage reported persistent acquired impairment of spoken or written language, or both, and they had damage to regions perfused by the left middle cerebral artery. Ischemic stroke due to vascular disease was the most common cause of damage, but other etiologies were represented, including hemorrhage due to aneurysm or AVM (9), gunshot wound (1), and herpes encephalitis (1). The average time post onset of aphasia was 3.5 years, with a range from 3 months to 36 years. Excluded from this cohort were individuals who were suspected of having pre-existing or progressive cognitive impairment suggesting dementia, and individuals who had co-occurring medical conditions that prevented reliable data collection.

**FIGURE 1:**
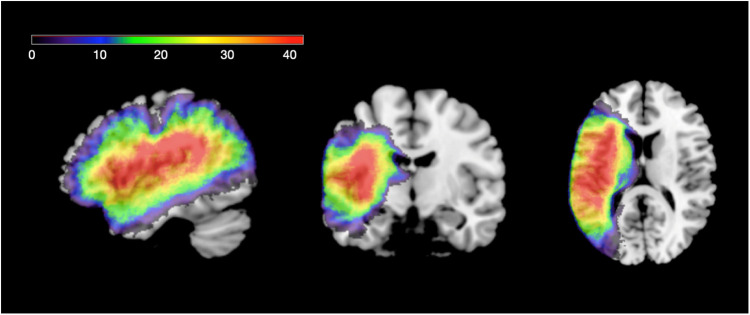
Lesion overlap for individuals with acquired language impairment.

To confirm lesion location, we initially reviewed clinical scans, and acquired high-resolution MRI brain scans whenever possible to precisely map the regions of damage. T-1 images were used as the primary source, with additional information gained from T-2 and FLAIR images. In the instances where magnetic resonance imaging was contraindicated, clinical CT scans were used to draw lesion maps in native space and interpolation was used to support warping to MNI space. Ultimately, scans were available and mapped for all but four individuals. Figure 1 depicts the overlap of the lesion maps from 83 of 87 individuals, showing common areas of damage centered in the left perisylvian region. The figure was generated from hand drawn lesions on brain images in native space and warped to the MNI template brain following procedures described by Andersen et al. (2010).

The lesion maps in Figure 1 are consistent with other group studies including individuals with left middle cerebral artery stroke. Damage extends throughout the entire left perisylvian region which includes the dorsal language pathway. Only a few individuals had damage that extended outside of the MCA regions, which occurred with hemorrhagic stroke and the one case of herpes encephalitis that damaged anterior temporal lobe structures.

Performance on behavioral measures was evaluated in relation to data collected from neurotypical adults with no significant history of developmental or acquired impairment of language, cognition, or sensorimotor abilities. They all passed the *Mini-Mental State Examination* (MMSE; Folstein et al., 1975) with scores between 28 and 30 (mean = 29.3, *SD* 0.80).

Table 1 provides a summary of participant demographic characteristics. As is typical in stroke populations, the language-impaired cohort averaged 61.9 years (range 23–82 years) and the ages of the neurotypical control group were comparable. Most participants were right-handed, but 21 individuals with left hemisphere brain damage shifted to use of the left hand due to right hemiparesis. The language-impaired group had a high proportion of males (73.6%) while the control group, which included many of their female partners, had a lower proportion of males (34.2%). Gender differences were not expected to influence behavioral performance, which was ultimately confirmed (see below). The racial/ethnic composition was comparable with both groups reporting about 86% as non-Hispanic Caucasian (Table 1). English was the primary language for all, with about 10% reporting a different first language, Spanish being most prevalent. Both cohorts were relatively well-educated, averaging 2–3 years of college. Participants rated their (premorbid) reading and spelling skills on a 1–5 scale (with 1 = poor and 5 = excellent), and the two groups showed comparable perceptions of above average skills (4+ out of 5) (see Table 1).

**TABLE 1:** Demographic characteristics of those with acquired language impairment (*n* = 87) and neurotypical controls (*n* = 38).

	Language impaired Mean (*SD*)	Controls Mean (*SD*)	Compare *P-value*
Age in years (*SD*)	61.9 (12.6)	62.8 (11.1)	0.724^1^
Sex (% Male)	73.6% (64M:23F)	34.2% (13M:25 F)	<0.001^2^
Right-Handed (premorbid) Changed to LH	92.0% (80RH; 6LH, 1Am) 21	86.8% (33RH; 5LH) N/A	0.509^2^
Education (years)	14.6 (2.9)	15.9 (2.7)	0.097^1^
Rating of Reading Skill (1–5)	4.7 (0.9)	4.4 (1.4)	0.682^1^
Rating of Spelling Skill (1–5)	4.8 (1.1)	4.5 (1.4)	0.788^1^
**Race/Ethnicity %**			
Asian American	0	2.6	
African American	0	0	
Hispanic	11.5	7.9	
Multiracial	1.1	2.6	
Native American	1.1	0	
White (Non-hispanic)	86.2	86.8	

^1^Group comparison using independent samples *t*-test.

^2^Significance test of independent proportions.

### Behavioral assessment

Before testing began, we affirmed adequate vision and hearing for completion of the tasks included in this study, allowing for visual correction (eyeglasses or contact lenses) and sound amplification as needed. Individual hearing status was determined by pure tone air-conduction thresholds or existing audiologic records, and hearing was within normal limits for 70 of 87 individuals with language impairment and 34 of 38 in the control group. For all others, we assured adequate hearing for behavioral testing using personal hearing aids or amplification via headphones provided by the examiner, and a quiet testing environment.

#### Characterizing spoken language profiles

For those with acquired brain damage, comprehension and production of spoken language was characterized by performance on the oral language portions of the *Western Aphasia Battery* (WAB; Kertesz, 1982), yielding aphasia quotients ranging from 14.1 to 98.8 (*M* = 63.3, *SD* = 23.9). Consistent with WAB scoring procedures, 10-point composite scores were derived for each individual to characterize auditory comprehension, spoken repetition, and naming ability, which complement the ratings for content and fluency. Aphasia types were determined in the standard manner, with the addition of a Borderline Fluent category to capture the typical profile of those who evolved from Broca’s aphasia toward Anomic aphasia (i.e., fluency ratings of 5 or 6). The distribution of aphasia types was as follows: 2 Global, 9 Wernicke’s, 11 Conduction, 21 Broca’s, 9 Borderline Fluent, 29 Anomic aphasia, and 6 who tested as minimally aphasic. The latter six individuals remained in the cohort because they reported persistent language impairment, and this was confirmed by the evaluation of spoken naming, reading, and spelling as detailed below.

Regarding speech production, mild dysarthria was present in some individuals, but not to the extent that it interfered with intelligibility. Evidence of impaired motor planning for speech was detected in 29 of the 87 with language impairment which warranted additional evaluation. Using a motor speech evaluation protocol consistent with Duffy (2005), the distribution of apraxia of speech severity was 9 mild, 5 moderate, 7 moderately severe, and 8 severe.

#### Primary dependent measures: Single-word naming, reading, and spelling

**FIGURE 2:**
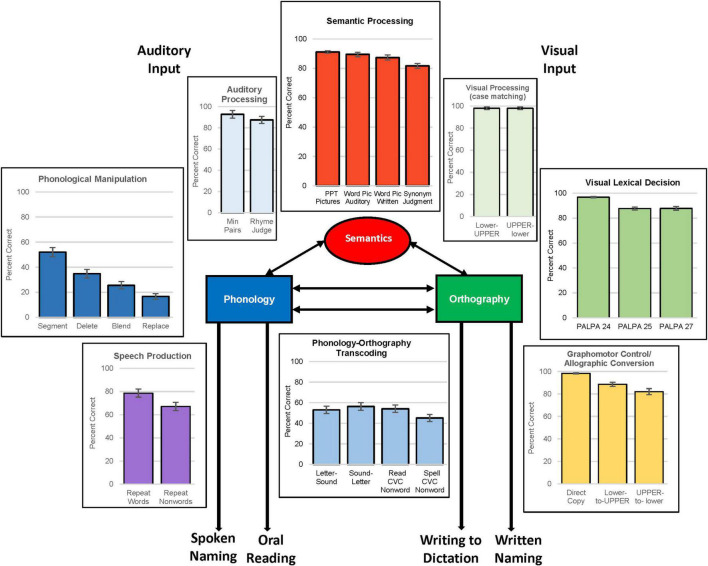
Schematic model of cognitive processes supporting spoken and written language processing with performance on representative tasks by language-impaired cohort.

As depicted in Figure 2, the primary dependent measures of interest for this study were the single-word tasks of spoken/written naming, oral reading, and writing to dictation. There was considerable range in performance on these tasks by individuals with acquired language impairment, and they performed below the neurotypical cohort on all tasks (Table 2). We confirmed that there were no significant gender differences on any of the tasks, as expected.

**TABLE 2:** Performance on single-word language measures of interest reported as percent correct, showing statistical comparison between language-impaired (LI) and neurotypical control (NC) groups.

Task (# items)	*n* LI, NC	Lang impaired Mean (*SD*)	Neurotypical Mean (*SD*)	Comparison *t, p-values*
**Picture Naming**				
Boston Naming Test (60)	87, 38	44.1 (32.6)	94.7 (4.2)	–14.17, <0.001
**Object Naming (from WAB)**				
Spoken Naming (20)	87, 28	62.2 (38.3)	100.0 (0.0)	–9.22, <0.001
Written Naming (20)	81, 28	46.1 (34.2)	99.5 (2.1)	–13.96, <0.001
**Oral Reading**				
Words (80)	87, 38	58.4 (40.3)¥	99.8 (0.5)	–9.58, <0.001
Regular (40)	87, 38	60.2 (41.0)§	99.8 (0.8)	–8.99, <0.001
Irregular (40)	87, 38	56.2 (40.2)	99.8 (0.6)	–10.14, <0.001
Non-words (20)	87, 38	29.4 (33.2)	98.0 (3.9)	–18.92, <0.001
**Written Spelling**				
Words (80)	87, 38	38.1 (35.8)¥	98.4 (2.0)	–15.70, <0.001
Regular (40)	87, 38	41.2 (38.4)§	99.8 (0.6)	–14.21, <0.001
Irregular (40)	87, 38	34.9 (33.9)	96.9 (4.0)	–16.70, <0.001
Non-words (20)	87, 38	22.1 (29.6)	96.8 (4.6)	–22.91, <0.001

¥, lexicality effect (words > non-words).

§, regularity effect (regular words > irregular words).

Spoken naming was measured using the *Boston Naming Test* (Kaplan et al., 2001). Mean raw scores out of 60 were 26.5 and 56.8 for the language-impaired and control groups, respectively. We also administered a written naming task using the 20 objects from the *Western Aphasia Battery* presented in the same manner as the spoken naming task of the WAB. For comparison between spoken and written naming of objects, responses were simply scored as correct or incorrect without partial credit for paraphasias or misspellings. As shown in Table 2, individuals with language impairment demonstrated significant word retrieval difficulties, and written naming was more impaired than spoken naming in that cohort, *t*(80) = 5.59, *p* < 0.001.

Reading and spelling of individual words were tested using the Arizona Battery for Reading and Spelling (ABRS) which includes 40 regularly spelled words, 40 irregularly spelled words, and 20 non-words that have been used to characterize alexia/agraphia profiles (e.g., Henry et al., 2007; Rapcsak et al., 2009; Beeson et al., 2010). Words range in length from 4 to 7 letters (*M* = 5.1, *SD* = 0.92), and the non-words are comparable in length (*M* = 4.95; *SD* = 0.74). Several linear mixed model analyses were conducted: first, to examine the effects of group and modality (reading vs. spelling), and then to examine lexicality (words vs. non-words) and regularity (regular vs. irregular) effects for reading and spelling, separately. As shown in Table 2, the language- impaired group was significantly impaired on all reading and spelling tasks in relation to controls; that is, there was a significant main effect of group for each of the analyses. Whereas the controls showed no difference between reading and spelling accuracy, those with language impairment were less accurate on spelling compared to reading words by 20.3%, *t*(121) = –4.1, *p* < 0.001. For both reading and for spelling, there were significant interactions for group × lexicality and group × regularity. Whereas the control group did not show any effects, those with language impairment had a significant lexicality effect in both reading and spelling, in that real words were better than non-words by 29% for reading, *t*(121) = –5.6, *p* < 0.001, and 16% for spelling, *t*(121) = –3.8, *p* < 0.001. Those with language impairment also showed a small, but significant, regularity effect in that regular words averaged 4% better than irregular words for reading, *t*(121) = –3.6, *p* < 0.001, and regular words were 6% better than irregular for spelling, *t*(121) = –1.99, *p* = 0.049.

We also evaluated individual reading and spelling performances to characterize alexia and agraphia profiles. To do so, the difference in percent correct for words versus non-words was calculated to obtain a lexicality measure for each individual for reading and spelling. Similarly, the percent correct for regular versus irregular word reading and spelling was calculated for a regularity measure. The magnitude of the lexicality and regularity effects for each person with language impairment were tested against the distribution of the neurotypical control group using the single-subject statistical approach of Crawford and Howell (1998). Those with a significant lexicality effect were characterized as demonstrating phonological alexia or agraphia. Those with a regularity effect and the presence of phonologically plausible errors were characterized as having surface alexia or agraphia. When fewer than 30% of responses were correct on real words, the profile was designated as global alexia or global agraphia, consistent with our previous work (Beeson and Henry, 2008; Rapcsak et al., 2009). Individual reading profiles were distributed as follows: 59% phonological alexia, 34% global alexia, 5% unclassifiable (no lexicality or regularity effect), and 2% were unimpaired. For spelling, the distribution was 51% phonological agraphia, 30% global agraphia, 6% were unclassifiable, and 3% had surface agraphia. These findings are consistent with previous studies demonstrating that damage to dorsal pathways is associated with phonological alexia/agraphia whereas damage to ventral (semantic) pathways are typically required to produce surface alexia/agraphia.

#### Potential predictor variables: Sensorimotor skills and central cognitive processes

The comprehensive behavioral assessment included evaluation of peripheral sensorimotor abilities necessary for spoken and written language as well as the underlying cognitive/linguistic skills that support language. As anticipated, the neurotypical group performed near ceiling on most language tasks and variance was limited. In Figure 2, we provide a simplified model of language processing and graphic display of average performance on tasks intended to characterize the status of component processes in the language-impaired group.

##### Sensorimotor skills

###### Auditory and visual processing

Several measures from the *Psycholinguistic Assessments of Language Processing in Aphasia* (PALPA; Kay et al., 1992) provided an index of the status of auditory and visual processing of language input. Language-impaired individuals demonstrated mild difficulty relative to controls on the auditory perception tasks of minimal pair judgment (PALPA 1 and 2) and rhyme judgment (PALPA 15) (see Table 3). These tasks required some phonological awareness but did not require explicit identification or manipulation of sublexical phonological information. There were no group differences between the language-impaired group and neurotypical controls on tasks that required visual recognition of letters presented in reverse orientation (PALPA 18) or matching upper to lowercase letters and vice versa (PALPA 19 and 20), suggesting preserved processing of visual input and recognition of letter shapes.

**TABLE 3:** Performance on comprehensive assessment battery by those with language impairment (LI) and neurotypical controls (NC) with group comparison statistics.

	*n* LI, NC	Language impaired Mean (*SD*)	Controls Mean (*SD*)	Comparison *t, p-values*
**Auditory Processing (Words)**				
Minimal Pairs PALPA 1 and 2 (yes-no)	86, 38	92.6 (10.5)	99.4 (1.5)	–5.83, <0.001
Rhyme Judgment PALPA 15 (yes-no)	86, 38	87.4 (15.4)	97.5 (2.9)	–5.83, <0.001
**Visual Processing (Letters)**				
Letter Reversal PALPA 18 (point)	87, 37	97.5 (7.3)	99.2 (2.2)	–1.33, 0.094
Case Matching (lower-upper) P19 (point)	87, 37	97.9 (11.0)	99.9 (0.6)	–1.09, 0.139
Case Matching (upper-lower) P20 (point)	87, 37	98.0 (11.0)	99.9 (0.1)	–1.04, 0.150
**Visual Problem-Solving**				
Raven’s Coloured Prog. Matrices (of 36)	87, 38	28.5 (6.3)	32.2 (3.4)	–4.123, <0.001
**Semantic Processing**				
Pyramids and Palm Trees* (pic) (point)	87, 38	91.2 (7.3)	98.4 (1.8)	–8.64, <0.001
Pyramids and Palm Trees (written) (point)	87, 30	84.7 (13.8)	98.2 (1.9)	–8.85, <0.001
Arizona Semantic Test (point)	87, 38	85.5 (15.6)	99.2 (1.2)	–8.15, <0.001
Spoken Word-Pic PALPA 47* (point)	87, 37	89.5 (14.3)	99.7 (0.8)	–6.61, <0.001
Written Word-Pic PALPA 48* (point)	87, 37	87.4 (16.0)	99.9 (0.4)	–7.31, <0.001
Synonym Judgment PALPA 49 (yes-no)	87, 36	81.7 (15.7)	98.7 (2.8)	–9.68, <0.001
**Phonological Tasks**				
Rhyme Production (spoken)	85, 30	59.2 (36.1)	98.7 (4.3)	–9.89, <0.001
Sound Segmentation (spoken)	87, 38	52.1 (33.8)	97.3 (4.2)	–12.26, <0.001
Sound Segmentation (write)	87, 38	51.8 (33.6)	98.8 (2.6)	–12.96, <0.001
Sound Deletion* (spoken)	87, 38	34.9 (31.8)	98.2 (2.9)	–18.40, <0.001
Sound Blending* (spoken)	87, 38	25.6 (27.3)	90.7 (10.1)	–19.36, <0.001
Sound Replacemen* (spoken)	87, 38	16.7 (21.1)	89.0 (8.8)	–27.05, <0.001
Digits Forward Span (raw score, spoken)	86, 38	3.3 (2.6)	9.6 (1.8)	–13.71, <0.001
Digits Forward Span (raw score, pointing)	86, 38	2.4 (2.6)	9.8 (1.5)	–19.92, <0.001
**Orthographic Recognition (Words)**				
Visual Lexical Dec PALPA 24 (mark)	87, 37	96.8 (4.5)	98.8 (4.1)	–2.30, 0.023
Visual Lexical Dec PALPA 25* (mark)	87, 38	87.7 (11.4)	98.9 (2.2)	–2.39, <0.01
Visual Lexical Dec PALPA 27* (mark)	87, 37	87.8 (13.3)	98.7 (1.5)	–7.52, <0.001
**Phonology-Orthography Transcoding**				
Letter-Sound (spoken)	87, 38	53.1 (33.8)	98.6 (2.8)	–12.45, <0.001
Sound-Letter (write)	87, 38	56.3 (34.9)	98.2 (4.3)	–11.02, <0.001
Reading CVC non-words (spoken)	87, 38	54.1 (33.2)	99.0 (1.8)	–12.58, <0.001
Spelling CVC non-words (write)	86, 38	45.1 (31.6)	98.3 (2.6)	–15.48, <0.001
**Speech Production**				
Repeat Words* (spoken)	87, 38	78.6 (32.4) ¥	99.6 (1.4)	–6.03, <0.001
Repeat Non-words* (spoken)	87, 38	67.1 (33.2)	96.7 (6.8)	–7.95, <0.001
Apraxia of Speech Rating* (0 = no AOS)	87, 38	0.84 (1.38)	0 (0)	5.67, <0.001
**Handwriting**				
Copy Words* (write)	87, 38	98.3 (5.5)	99.9 (0.3)	–1.76, 0.041
Lower-uppercase Conversion* (write)	87, 36	88.6 (17.6)	99.3 (2.2)	–3.60, <0.001
Upper-lowercase Conversion* (write)	87, 36	82.0 (25.2)	99.3 (1.5)	–6.32, <0.001

*Tests included in trimmed principal components model.

¥, lexicality effect (words > non-words).

To provide an index of cognitive ability apart from language processing, the *Raven’s Coloured Progressive Matrices* (Raven, 1977) was administered to test non-verbal (visual) problem solving. The majority of those with language impairment (59 of 87) performed within the range of the control group, but 28 fell below expectations (<26 of 36 correct, determined using methods from Crawford and Howell, 1998). As a group, the language-impaired cohort performed significantly below the control group, *t*(122) = –4.03, *p* < 0.001 (Table 3).

###### Speech production

To characterize speech production ability, we examined spoken repetition of 20 single words and 20 non-words taken from the Arizona Battery for Reading and Spelling and matched for length in letters, sounds, and number of syllables. As a group, those with language impairment performed below controls (Table 4), yet at the individual level, about 45% (*n* = 39) of those with language impairment were able to repeat words without error. Those who had marked difficulty with single word repetition included individuals with moderately severe or severe apraxia of speech (*n* = 15) as well as individuals with conduction or Wernicke’s aphasia where phonological assembly difficulties were apparent. In those with aphasia, real words were repeated better than non-words by 11.6%, *t*(123) = –3.402, *p* < 0.001 (Table 3). As noted earlier, lexicality effects have been interpreted to reflect central phonological impairment that disproportionately affects non-words because these novel items contain unfamiliar combinations of phonological elements that are more difficult to process. In addition, unlike real words, non-words cannot derive top–down support from semantic representations. However, the unfamiliarity of non-words may also make it more difficult to map phonological units onto the appropriate motor representations during speech production/articulatory coding. Thus, lexicality effects in spoken repetition may also be attributable, at least in part, to a peripheral processing impairment.

**TABLE 4:** Results from omnibus principal components analysis implemented using 33 test scores from 85 individuals with language impairment.

	Component	1	2	3	4	5
						
		Phon	Semantic	Speech	Allog	Visual
1	Spell Non-words (write)	**0.89**	0.06	0.16	0.06	0.16
2	Sound Replacement (spoken)	**0.83**	0.08	0.22	0.07	0.16
3	Spell Regular Words (write)	**0.83**	0.20	0.26	0.16	0.29
4	Read Non-words (spoken)	**0.82**	0.17	0.23	0.12	0.22
5	Spell CVC Non-words (write)	**0.82**	0.34	0.22	0.11	0.15
6	Spell Irregular Words (write)	**0.77**	0.21	0.24	0.17	0.35
7	Sound Segmentation (written)	**0.74**	0.39	0.27	0.23	0.14
8	Sound Deletion (spoken)	**0.73**	0.34	0.27	0.10	0.08
9	Transcode Sound-Letter (write)	**0.71**	0.46	0.26	0.18	0.02
10	Digit Span Forward (point)	**0.66**	0.16	0.36	0.24	0.04
11	Read CVC Non-words (spoken)	**0.62**	0.41	0.49	0.09	0.16
12	Sound Blending (spoken)	**0.62**	0.28	0.29	0.14	–0.05
13	Transcode Letter-Sound (spoken)	**0.57**	0.41	0.25	0.07	–0.17
14	Sound Segmentation (spoken)	**0.50**	0.42	0.44	0.10	–0.03
15	AZ Semantic Test (point)	0.20	**0.80**	0.02	0.28	0.09
16	Written Word-Picture P48 (point)	0.29	**0.78**	0.27	0.09	0.16
17	Pyramids Palm Trees pics (point)	0.10	**0.77**	0.03	0.35	0.19
18	Spoken Word-Picture P47 (point)	0.32	**0.73**	0.26	–0.08	–0.02
19	Synonym Judgment P49 (yes-no)	0.31	**0.70**	0.26	–0.05	0.23
20	Repeat Words (spoken)	0.24	0.20	**0.85**	0.03	0.07
21	Repeat Non-words (spoken)	0.31	0.14	**0.83**	0.10	0.04
22	Apraxia Rating (spoken)^1^	0.16	0.11	**0.68**	–0.10	–0.12
23	Digit Span Forward (spoken)	0.45	–0.08	**0.67**	0.02	0.05
24	Read Irregular Words (spoken)	0.47	0.42	**0.65**	0.02	0.26
25	Read Regular Words (spoken)	0.48	0.42	**0.63**	0.04	0.25
26	Boston Naming Test (spoken)	0.46	0.50	**0.54**	–0.05	0.21
27	Upper-Lowercase Letter (write)	0.35	0.29	–0.03	**0.78**	0.02
28	Ravens Colored Prog M (point)	0.14	0.15	0.05	**0.76**	0.13
29	Direct copy words (write)	0.00	–0.15	0.04	**0.75**	0.06
30	Lower-Uppercase Letter (write)	0.31	0.33	–0.19	**0.71**	0.09
31	Visual Lex Decision P24 (mark)	0.02	0.06	0.15	**0.58**	0.49
32	Visual Lex Decision P25 (mark)	0.32	0.10	0.03	0.14	**0.81**
33	Visual Lex Decision P27 (mark)	0.21	0.28	0.00	0.21	**0.75**
	Initial Eigenvalue	16.33	3.29	2.12	1.54	1.38
	% Variance (rotated model)	27.91	15.66	14.85	9.57	6.70
	Cumulative % Variance (rotated model)	27.91	43.57	58.42	67.99	74.69

Factor scores greater than 0.50 in bold.

Components: 1 = phonological skill, 2 = semantic processing, 3 = speech production, 4 = allographic skill, 5 = visual orthographic recognition.

Extraction method: Principal component analysis after varimax rotation with Kaiser normalization.

Rotation converged in six iterations. Kaiser–Meyer–Olkin measure of sampling adequacy = 0.889.

Bartlett’s test of sphericity = 3081.23, *df* = 528, *p* < 0.001.

^1^Scale direction converted (lower = more impaired).

###### Handwriting

A direct copying task using 10 common words affirmed that all participants had adequate motor control of the hand to generate legible letter shapes, and there was no evidence of apraxic agraphia. The ability to recall and produce individual letter shapes was sampled using case conversion tasks that required generation of uppercase letters in response to visual presentation of lowercase forms and vice versa. As a group, those with aphasia were impaired on these case conversion tasks relative to the neurotypical controls (Table 3). At the individual level, about half of the language-impaired cohort performed these tasks within the normal range, while those who performed more poorly than controls ranged from 0 to 92% correct.

##### Central cognitive processes that support language performance

###### Semantic processing

Five measures of semantic knowledge were administered: the spoken word-to-picture matching and written word-to-picture matching tasks from the PALPA (Subtests 47 and 48), and an auditory synonym judgment task (PALPA 49). We also administered the picture version of the *Pyramids and Palm Trees Test* (Howard and Patterson, 1992) and the 40-item *Arizona Semantic Test* that is similar to the *Camels and Cactus Test* in that it includes four options to match the presented picture (rather than the forced choice from two items on the PPT). As shown in Table 3, there was significant impairment on each of these measures in relation to the neurotypical group.

###### Phonological skills

Phonological abilities were examined using tasks that required the identification, maintenance, and manipulation of sublexical phonology in the context of both word and non-word stimuli. The tasks did not involve presentation of orthographic stimuli. For those with language impairment, rhyme production (“Say something that rhymes with *five.*”) was significantly impaired (59% correct), as was performance on a sound segmentation task (“Say the first/last sound in ‘teb”’; 52.1% correct). Three additional phonological manipulation tasks revealed increasing difficulty: sound deletion (e.g., “Say *foap*, now take away the /f/”), sound blending (e.g., “Put these sounds together, /s/ /i/ /b/”) and sound replacement (e.g., “Say *goob*, now change the /g/ to /m/”). Sound blending and replacement presented some challenge to the control group which typically averaged > 97% correct on assessment measures (see Table 3). On sound blending, controls averaged 90.7% correct compared to 25.6% in the language-impaired group; on sound replacement the contrast was 89% for controls and 16.7% for those with language impairment.

The digits forward subtest from the *Wechsler Memory Scale-Revised* (WMS-R; Wechsler, 1987) was administered to provide an index of phonological short-term memory, and an analogous auditory span task was administered that required pointing to a sequence of spoken digits without a verbal response. The language-impaired cohort was significantly impaired on these with an average span of 3–4 digits on the WMS-R forward span task, and an average of 3 digits on the comparable pointing task as compared to the control average span of 7 for both versions of the task.

###### Orthographic processing

Three lexical decision subtests from the PALPA were administered to examine visual word recognition. Although lexical decision tasks are often considered to be pure orthographic tasks, there is evidence that they automatically engage phonological and semantic representations. In fact, it has been proposed that the VWFA constitutes a critical neural interface between spoken and written language and plays an integrative role that involves both bottom–up and top–down interactions among orthographic, semantic, and phonological representations during reading (Price and Devlin, 2011) and spelling (Rapcsak and Beeson, 2015). In our cohort, there were no group differences on the easiest task (PALPA 24) which contrasts real words with implausible letter strings, but the language-impaired group performed significantly below the control group on the visual lexical decision tasks with foils that include word-like letter strings (PALPA 25 and 27). Given that our cohort had adequate vision and anatomically spared VWFA, impaired performance on these orthographic lexical processing tasks is likely attributable to weakened input from semantic and phonological representations.

###### Orthography-phonology relations

Whereas the tasks above were intended to isolate domain-specific skills, we also assessed orthography-phonology transcoding skills that are fundamental to reading and spelling. Transcoding tasks included producing individual letter-sound (spoken) and sound-letter (written) correspondences for 20 single consonants, as well as reading and spelling of consonant-vowel-consonant (CVC) non-words (e.g., *meb*). The CVC non-word reading and spelling tasks were comparable to the ABRS non-word reading and spelling but were more sensitive to transcoding skills as they were scored sound-by-sound or grapheme-by-grapheme (rather than binary correct/incorrect). As shown in Table 3, those with language impairment had marked difficulty with transcoding tasks compared to the control group.

### Planned statistical analyses

Several exploratory PCA were planned using assessment measures with the intention of identifying independent factors that account for variation in participant performance. To allow comparison with previous research, we conducted analyses with the language-impaired group alone, but also implemented parallel analyses with the neurotypical controls included. Initial omnibus PCAs were inclusive of the primary dependent measures (naming, reading, and spelling tasks), which were removed for a second set of PCAs, as we aimed to evaluate the prediction of those measures. A final, trimmed PCA model was implemented with the goal of retaining tasks that were relatively pure measures of the latent variables that emerged from the initial, more comprehensive PCA models. Consistent with previous research examining underlying impairments in phonological alexia/agraphia, we retained non-orthographic measures of phonological skill rather than tasks that require transcoding between sounds and letters (Patterson and Marcel, 1992; Rapcsak et al., 2009; Madden et al., 2018). We also removed measures that did not load as strongly on the emergent factors and to remove redundancy in an effort to approximate a relatively efficient clinical assessment protocol.

Factor scores derived from the final (optimal) PCA model were entered into multiple linear regression models to test prediction models for single-word language tasks that involve naming, reading, and writing in those with language impairment. To control for potential influence of education, weighted least squares regression models were implemented. The resulting standardized beta coefficients from regression equations were examined to evaluate the relative contributions of the factors. To provide broader context to interpret the predictive value of the factor scores, we also conducted linear regression analyses for the WAB aphasia quotient and the 10-point composite scores for comprehension, repetition, and naming.

## Results

### Principal components analyses: Deriving underlying factors

For the initial omnibus PCA, there were complete datasets for 35 variables from 85 of the 87 individuals with language impairment (LI) and 35 of 38 neurotypical controls (NC). Two tasks were excluded from further analysis (auditory rhyme judgment and auditory minimal pairs) due to weak factor loadings (<0.504), retaining 33 variables with significant loadings on one or more factors. Data from individuals with language impairment yielded a five-factor solution that accounted for 74.69% of the total variance (see Table 4). The factor loadings reflected the following constructs (and variance proportions): (1) phonological skills (27.9%), (2) semantic processing (15.7%), (3) speech production (14.9%), (4) allographic skill for writing letters (9.6%), and (5) visual orthographic processing (6.7%). All factors had initial eigenvalues greater than 1 and the scree plot inflection point was consistent with inclusion of five factors. Sampling adequacy was verified by the Kaiser–Meyer–Olkin measure (KMO = 0.93). As shown in Table 4, the phonological skill factor had strong loadings from single-word spelling, phonological manipulation tasks, as well as sound-letter and letter-sound transcoding and non-word reading/spelling tasks. This factor was distinct from a speech production factor with significant loadings from spoken repetition of words/non-words, the apraxia of speech rating, as well as oral reading of words, spoken digit span, and spoken naming. The tasks intended to assess semantics (e.g., spoken/written word to picture matching) all loaded strongly on a common semantic factor. Spoken naming also loaded on semantics, but to a lesser extent than speech production. The fourth factor included strong loadings from the allographic case conversion tasks (e.g., writing lowercase letter in response to uppercase letters) as well as the *Raven’s Coloured Progressive Matrices*. Finally, the fifth factor reflected performance on the visual lexical decision tasks only.

Outcomes from the same PCA implemented with 120 participants (85 LI, 35 NC) using scores on the 33 tasks yielded the same five-factor solution, accounting for 80.97% of the total variance (see Supplementary Table A). Factor loadings were similar to those shown in Table 4, with phonological skill accounting for 33.8% of the total variance.

The second pair of PCAs were conducted with data from 26 measures after the exclusion of naming (BNT), reading (regular, irregular, non-word), and spelling (regular, irregular, non-word) tasks (see Table 5). This PCA accounted for 73.1% of the variance when restricted to those with language impairment, and 79.3% of the variance when the controls were included (see Supplementary Table B). Sampling adequacy was verified by the Kaiser–Meyer–Olkin measure for the subset with language impairment (*n* = 85; KMO = 0.86) and for the larger cohort (*n* = 120; KMO = 0.91). Consistent with the omnibus PCA, the same five factors emerged with phonological skill accounting for the greatest proportion of variance at 25 and 28.5% for the language-impaired and full cohort, respectively.

**TABLE 5:** Results from principal components analysis implemented using 26 scores from 85 language-impaired individuals.

	Component	1	2	3	4	5
						
		Phon	Semantic	Speech	Allog	Visual
1	Sound Replacement (spoken)	**0.83**	0.04	0.18	0.07	0.26
2	Sound Deletion (spoken)	**0.80**	0.27	0.19	0.08	0.18
3	Spell CVC Non-words (write)	**0.76**	0.35	0.22	0.12	0.20
4	Sound Segmentation (written)	**0.73**	0.37	0.23	0.24	0.18
5	Digit Span Forward (point)	**0.72**	0.11	0.30	0.23	0.12
6	Transcode Sound-Letter (write)	**0.72**	0.45	0.23	0.19	0.04
7	Sound Blending (spoken)	**0.71**	0.21	0.20	0.11	0.04
8	Transcode Letter-Sound (spoken)	**0.66**	0.36	0.17	0.03	–0.08
9	Read CVC Non-words (spoken)	**0.63**	0.41	0.44	0.11	0.16
10	Sound Segmentation (spoken)	**0.61**	0.36	0.35	0.05	0.09
11	AZ Semantic Test (point)	0.26	**0.78**	–0.03	0.26	0.11
12	Written Word-Picture P48 (point)	0.32	**0.78**	0.23	0.09	0.14
13	Pyramids Palm Trees pics (point)	0.12	**0.77**	0.00	0.36	0.17
14	Spoken Word-Picture P47 (point)	0.36	**0.73**	0.23	–0.08	–0.02
15	Synonym Judgment P49 (yes-no)	0.33	**0.70**	0.24	–0.06	0.25
16	Repeat Words (spoken)	0.29	0.22	**0.84**	0.02	0.09
17	Repeat Non-words (spoken)	0.38	0.15	**0.81**	0.08	0.08
18	Apraxia Rating (spoken)^1^	0.20	0.15	**0.69**	–0.11	–0.09
19	Digit Span Forward (spoken)	0.49	–0.09	**0.63**	0.03	0.05
20	Direct copy words (write)	–0.06	–0.10	0.08	**0.77**	0.03
21	Upper-Lowercase Letter (write)	0.38	0.26	–0.07	**0.77**	0.06
22	Ravens Colored Prog M (point)	0.17	0.12	0.00	**0.75**	0.15
23	Lower-Uppercase Letter (write)	0.32	0.30	–0.23	**0.71**	0.10
24	Visual Lex Decision P24 (mark)	–0.05	0.11	0.19	**0.58**	**0.51**
25	Visual Lex Decision P25 (mark)	0.25	0.10	0.04	0.13	**0.85**
26	Visual Lex Decision P27 (mark)	0.20	0.25	–0.04	0.18	**0.80**
	Initial Eigenvalue	11.53	3.13	1.81	1.30	1.23
	% Variance (rotated model)	24.99	16.26	12.31	11.71	7.81
	Cumulative % Variance (rotated model)	24.99	41.25	53.56	65.27	73.08

Factor scores greater than 0.50 in bold.

Components: 1 = phonological skill, 2 = semantic processing, 3 = speech production, 4 = allographic skill, 5 = visual orthographic recognition.

Extraction method: Principal component analysis after varimax rotation with Kaiser normalization.

Rotation converged in seven iterations. Kaiser–Meyer–Olkin measure of sampling adequacy = 0.855.

Bartlett’s test of sphericity = 1863.59, *df* = 325, *p* < 0.001.

^1^Scale direction converted (lower = more impaired).

We applied several criteria to further trim and refine the PCA in an effort to retain the strongest unambiguous measures of the five latent factors that emerged from the initial analyses. For tasks that loaded on phonological skill, the sound-letter and letter-sound transcoding tasks and the reading/spelling of CVC non-words were removed in favor of non-orthographic phonological manipulation tasks (i.e., sound deletion, blending, replacement). We also removed the digit span tasks as they do not require phonological manipulation, and they place additional demands on phonological working memory. With regard to semantics, we excluded the Arizona Semantic Test in favor of the more commonly used *Pyramids and Palm Trees* picture test, although we note that performance on these two tests were comparable. We also removed the PALPA 49 Synonym Judgment task which was not as strong as the spoken/written word to picture matching tasks (PALPA 47 and 48). We retained the three tasks that loaded strongly on the speech production factor: repetition of words and non-words, and the apraxia of speech rating. Of the tasks that loaded on allographic skills, we kept the written word copying task and the two case conversion tasks, but removed the *Ravens Coloured Progressive Matrices* test. Although the RCPM scores aligned with the letter production tasks which similarly require visuospatial attention, this test includes a complex visual problem-solving component that is distinct from the handwriting tasks. Regarding visual processing, we retained the PALPA 25 and 27 lexical decision tasks, but excluded the relatively easy PALPA 24 lexical decision as it had weak loadings that straddled the allographic and visual processing factors. In summary, fourteen tasks were retained for the final PCA as listed in Table 6. This included three tasks that had loaded strongly on the previously determined factors, except for visual processing with only two tasks retained.

**TABLE 6:** Results from optimal principal components analysis implemented using 14 scores from 87 language impaired individuals.

	Component	1	2	3	4	5
						
		Speech	Phon	Semantic	Allog	Visual
1	Repeat Words (spoken)	**0.88**	0.24	0.21	0.01	0.10
2	Repeat Non-words (spoken)	**0.84**	0.31	0.15	0.05	0.10
3	Apraxia Rating (spoken)^1^	**0.80**	0.12	0.09	–0.04	–0.04
4	Sound Replacement (spoken)	0.22	**0.85**	0.11	0.07	0.25
5	Sound Deletion (spoken)	0.25	**0.80**	0.30	0.10	0.19
6	Sound Blending (spoken)	0.27	**0.78**	0.21	0.15	0.03
7	Written Word-Picture P48 (point)	0.32	0.21	**0.82**	0.10	0.20
8	Spoken Word-Picture P47 (point)	0.28	0.27	**0.79**	–0.06	0.01
9	Pyramids Palm Trees pics (point)	0.01	0.11	**0.78**	0.30	0.19
10	Direct copy words (write)	0.05	–0.07	–0.15	**0.83**	0.04
11	Upper-Lowercase Letter (write)	0.01	0.28	0.31	**0.78**	0.17
12	Lower-Uppercase Letter (write)	–0.08	0.22	0.38	**0.73**	0.19
13	Visual Lex Decision P25 (mark)	0.10	0.17	0.07	0.11	**0.90**
14	Visual Lex Decision P27 (mark)	–0.01	0.16	0.22	0.17	**0.85**
	Initial Eigenvalue	5.60	2.26	1.23	1.16	0.93
	% Variance (rotated model)	17.86	17.45	17.35	14.38	12.79
	Cumulative % Variance (rotated model)	17.86	35.31	52.66	67.04	79.83

Factor scores greater than 0.50 in bold.

Components: 1 = speech production, 2 = phonological skills, 3 = semantic processing, 4 = allographic skill, 5 = visual orthographic recognition.

Extraction method: principal component analysis after varimax rotation with Kaiser normalization.

Rotation converged in six iterations. Kaiser–Meyer–Olkin measure of sampling adequacy = 0.776.

Bartlett’s test of sphericity = 740.52, *df* = 91, *p* < 0.001.

^1^Scale direction converted (lower = more impaired).

The PCA derived from the fourteen assessment measures from all 87 individuals with language impairment resulted in a strong model, accounting for 79.8% of the total variance (Table 6). Sampling adequacy was verified by the Kaiser–Meyer–Olkin measure (KMO = 0.78). We specified a 5-factor solution after varimax rotation, which was consistent with scree plot inflection. Initial eigenvalues were greater than 1.0 for all factors except visual processing, which was 0.93. The factor scores were consistent with previous PCAs performed with the larger data sets (compare to Tables 4, 5), but this trimmed model resulted in relatively equal proportion of variance accounted for by the first three factors: speech production (17.9%), phonological skill (17.5%, and semantics (17.4%). Allographic skill accounted for 14.4% of the total variance, followed by visual processing (12.8%). The comparable PCA conducted with data from all participants showed a similar five-factor solution accounting for 84% of the variance (Supplementary Table C). Again, the first three factors were relatively comparable regarding variance distribution: speech production (19.2%), semantic processing (18.5%) and phonological skill (18.3%), followed by allographic skill (14.3%) and visual processing (13.7%).

### Relative severity of impairments

**FIGURE 3:**
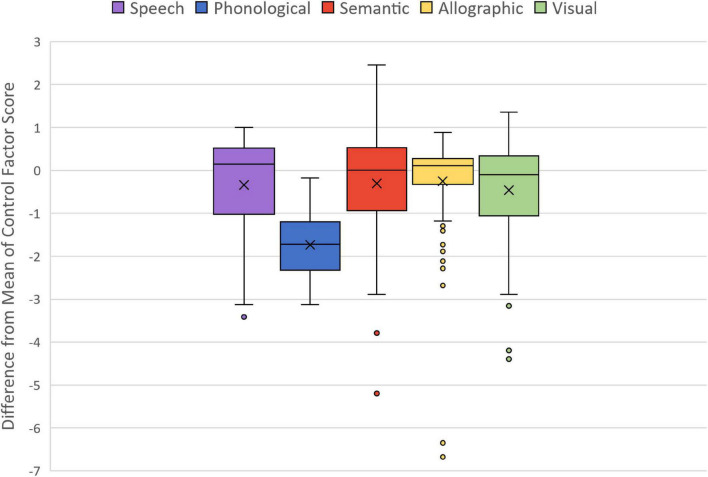
Box plots showing the distribution of factor scores as they differed from control means. Box boundaries are first and third quartiles, x = median.

Factor scores derived from the final PCA model were evaluated to characterize the relative performance levels of those with language impairment in relation to neurotypical controls. To do so, difference scores were calculated for each language-impaired individual in relation to the control mean for the five factors. The distribution of the difference scores depicted by the box plot in Figure 3 shows that phonological skills were more impaired than the other four factors. In fact, the other factors showed overlap with the control group (0 on the horizontal axis) indicating that some individuals performed within the normal range on all factors except that reflecting phonological skill. Figure 3 also shows the distribution of difference scores, which was relatively compact for phonological skill, and identified some outlier scores in semantic, allographic skill, and visual orthographic processing. These scores were reviewed to affirm accuracy and to discern potential explanatory factors on the basis of lesion location. The individual with the lowest semantic factor was the one case of language impairment acquired after herpes encephalitis that severely damaged left anterior temporal lobe and extended into the perisylvian region. One other individual with low semantic scores had vascular damage that extended to left anterior temporal cortex. A review of the outliers with marked allographic impairment showed that most had cortical damage that extended to left intraparietal sulcus/superior parietal lobule, regions at the periphery of left MCA. The one outlier with poor visual skills had a large frontal lesion.

### Linear regression models: Predicting performance on naming, reading, and spelling

Factor scores generated from the final (trimmed) PCA from the individuals with language impairment were entered as predictor variables in multiple linear regression analyses for the single-word language tasks of naming, reading, and spelling. For consistency, all five factors were entered into each prediction model, even though tasks varied regarding output modality (spoken or written).

**FIGURE 4:**
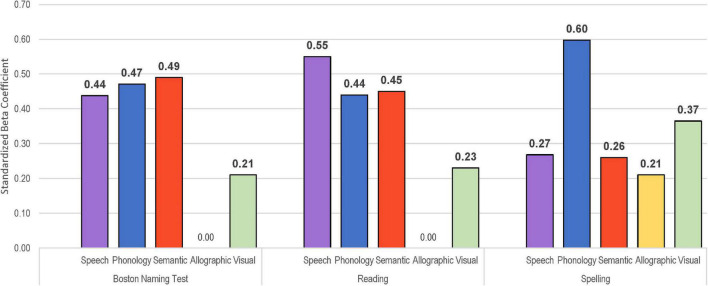
Visual depiction of standardized beta coefficient values for each of five factor scores from the multiple linear regression models to predict naming **(Left)**, reading single words aloud **(Middle)**, and writing words to dictation **(Right)**. Significant coefficients in bold.

**FIGURE 5:**
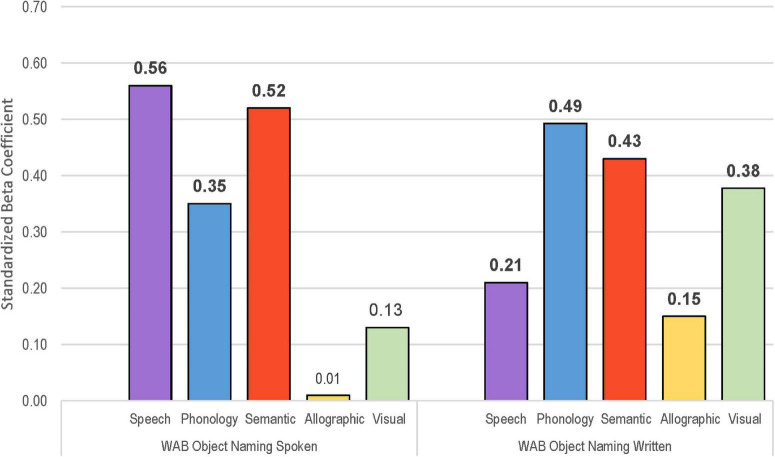
Visual depiction of standardized beta coefficient values for each of five factor scores from the multiple linear regression models to predict spoken object naming **(Left)** and written object naming **(Right)**, using stimuli from the WAB. Significant coefficients in bold.

**FIGURE 6:**
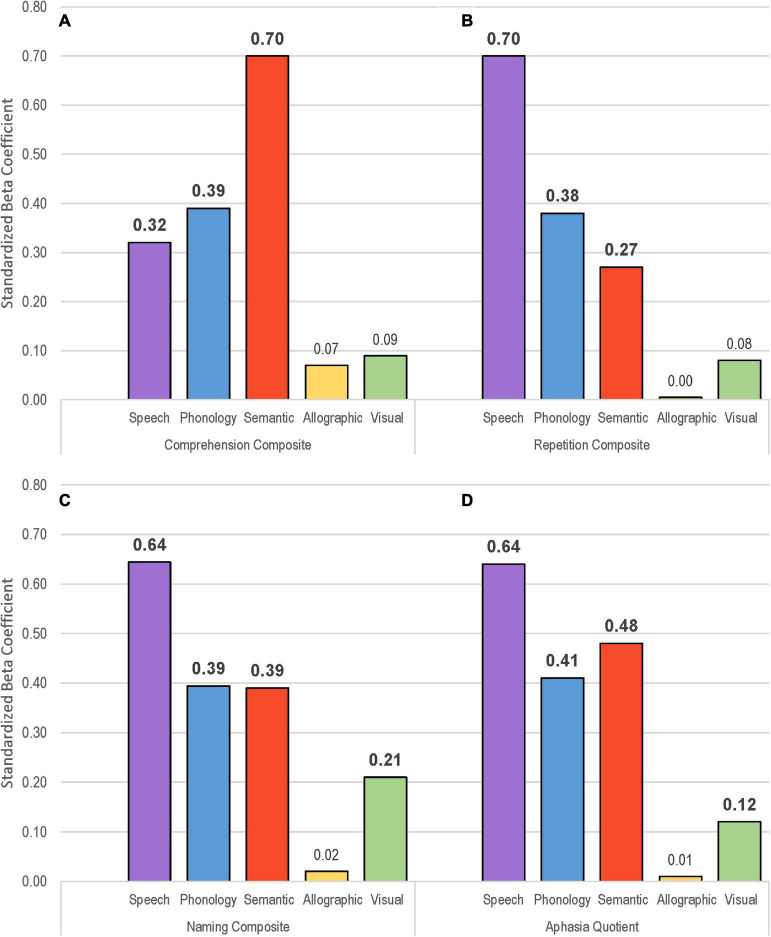
Visual depiction of standardized beta coefficient values for each of five factor scores from the multiple linear regression models to the following scores from the Western Aphasia Battery: comprehension composite **(A)**, repetition composite **(B)**, naming composite **(C)**, and aphasia quotient **(D)**. Significant coefficients in bold.

As shown in Table 7, all models were highly significant (*p* < 0.001) for the prediction of the following language measures: *Boston Naming Test*, oral reading of single words, written spelling to dictation, as well as oral and written naming of the objects from the WAB. The outcomes are described below for each of the variables with an emphasis on the relative weight of the predictor variables. For ease of comparison, the standardized beta coefficients were plotted for each of the factor scores across the tasks (Figures 4–6).

**TABLE 7:** Summary of multiple linear regression models using factor scores derived from principal components analysis to predict performance on selected language measures.

Language task	*R*	*R* ^2^	Adjusted *R*^2^	df1, df2	*F*	*P-value*
**Single-Word Tasks**						
Boston Naming Test	0.850	0.722	0.705	5, 81	42.099	<0.001
Read Words	0.877	0.769	0.755	5, 81	53.908	<0.001
Write Words	0.831	0.691	0.672	5, 81	36.289	<0.001
Object Naming: Spoken	0.862	0.743	0.727	5, 81	46.898	<0.001
Object Naming: Written	0.779	0.607	0.580	5, 75	23.122	<0.001
WAB Aphasia Quotient	0.916	0.839	0.829	5, 81	84.395	<0.001
Comprehension Composite	0.883	0.780	0.767	5, 81	57.500	<0.001
Repetition Composite	0.854	0.729	0.712	5, 81	43.609	<0.001
Naming Composite	0.876	0.768	0.754	5, 81	53.679	<0.001

The multiple linear regression model to predict performance on the BNT using derived factor scores accounted for 72.2% of the variance (Table 7). As depicted in Figure 4 (left), the standardized beta coefficients showed highly significant contributions from semantics, phonological skill, and speech production abilities. There was a small contribution from the visual factor, and allographic skill was not a significant predictor of spoken naming, as expected. The model to predict oral reading of single words was similarly strong, accounting for 76.9% of the variance, with speech production, semantics, and phonological skill all making significant contributions as shown in Figure 4 (middle). Visual processing was significant, but to a lesser extent, and allographic skill was not relevant for reading. Regarding writing words to dictation, the factor scores provided a strong model, accounting for 69.1% of the variance, with significant contributions from all factors (Figure 4, right). The contribution from phonological skills was particularly strong for the writing to dictation task (β = 0.60), and the contributions from visual processing and allographic skill were stronger on this task than for naming or oral reading (β = 0.37 vs. 0.21/0.23). The weight of contribution from speech production was noticeably less for the writing task (β = 0.27) than for naming and reading which required spoken output (β = 0.44/0.55). Semantic weight was also less for writing to dictation (where the spoken target word is provided) compared to spoken naming and oral reading that require word recall (β = 0.26 vs. 0.49/0.45).

Factor scores predicted spoken and written naming of WAB objects, accounting for 74.3 and 60.7% of the variance, respectively (Table 7). The spoken modality had large beta coefficients for semantics and speech production, along with phonological skill (Figure 5, left). Written naming showed strong contribution from phonological skill as well as semantics, visual processing, speech production, and allographic skill (Figure 5, right). As might be expected, semantic processing contributed more to written naming compared to writing to dictation (β = 0.43 vs. 0.26). The contribution from speech production was significant for written naming (β = 0.21) and writing to dictation (β = 0.27), but markedly less than tasks that required spoken naming (β = 0.56 for object naming, β = 0.44 for BNT).

Factor scores were also significant predictors of oral language performance as measured by the 10-point composite scores from the WAB for comprehension, repetition, and naming, as well as the aphasia quotient (Table 7). The relative weight of the standardized beta coefficients varied across the WAB measures in a manner consistent with the nature of the tasks (Figure 6). The regression model for comprehension composite scores accounted for 78% of the variance, and the semantic factor had the strongest beta coefficient (β = 0.70) as would be expected (see Figure 6A). The model for the repetition composite was also strong, accounting for 72.9% of the variance, with the speech production factor logically having the greatest weight (β = 0.70) (Figure 6B). The model for the naming composite similarly accounted for 76.8% of the variance, with the speech production factor having the strongest weight (β = 0.64, Figure 6C). The prediction model for the aphasia quotient accounted for 83.9% of the variance, with strong weights from speech production, semantics, and phonological skill (Figure 6D).

## Discussion

In this study, we examined the nature of spoken and written language impairments in a large group of individuals with damage in left middle cerebral artery territory affecting perisylvian speech/language areas that comprise the dorsal language pathway. Analysis of scores from the comprehensive assessment battery yielded strong principal component models with five factors that were consistent regardless of whether datasets included neurotypical controls or only those with language impairment. Examination of factor loadings from the behavioral measures yielded the following component descriptors: semantics, phonological skill, speech production, allographic skill, and visual orthographic processing. The relative importance of these factors was further clarified by the linear regression models predicting performance on word-level tasks (spoken and written naming, oral reading, and written spelling), as well as composite scores from the *Western Aphasia Battery* that reflect a range of spoken language tasks.

### An integrated model of spoken and written language processing

The five-component model derived from this study extends previous work that focused on spoken language data from similar cohorts which consistently identified semantic and phonological factors (Lambon Ralph et al., 2002; Butler et al., 2014; Mirman et al., 2015a; Thye and Mirman, 2018; Tochadse et al., 2018; Woollams et al., 2018). Our data revealed a comparable semantic factor, but rather than a single “phonological” factor, there was a clear separation of central “phonological skill” from the more peripheral processes that support speech production. An additional component captured orthographic processing critical to written language processing (reading and spelling). Finally, an allographic factor reflected peripheral skills for handwriting. Thus, the emergent model reflected the three central constructs of a triangle model of language processing (semantics, phonology, and orthography) and two peripheral components for the respective output modalities (speech production and handwriting), consistent with the model in Figure 2. Of these constructs, phonological skill showed the greatest impairment in our cohort.

Semantics, phonological skill, and speech production were all strong predictors of spoken naming and oral reading. The relative weight of the semantic factor was logically greater on naming tasks in contrast to oral reading, as the former is more dependent on concept retrieval. Similarly, the semantic factor was stronger in predicting written naming compared to writing to dictation. The semantic construct was further validated by the fact that it accounted for the greatest proportion of the variance in the auditory comprehension composite score of the WAB.

The phonological skill factor that emerged from the data reflected performance on non-orthographic tasks requiring isolation and manipulation of sounds (e.g., sound blending, deletion, replacement), as well as tasks that map sounds to letters and vice versa, and digit span tasks (spoken and pointing), when such tasks were included in the PCA. Thus, phonological skill was not specific to tasks with spoken output, and it was a significant predictor of performance across modalities: spoken and written naming, oral reading, and writing to dictation. In fact, this phonological factor was the strongest predictor of performance on written tasks where no overt speech production was required. These findings support a central phonological processing construct that is distinct from peripheral support for speech production. Although this separation is consistent with models of language processing, previous PCA models have rarely teased aspects of speech production from phonology (Lambon Ralph et al., 2002; Butler et al., 2014; Halai et al., 2017; Woollams et al., 2018).

The speech production factor distilled from our PCA was reflective of performance on the relatively straightforward task of spoken repetition of words and non-words. In our cohort, the repetition tasks captured impairments of motor control for speech as well as speech production difficulties related to phonological assembly (i.e., paraphasic errors). The former was confirmed by the loading of the apraxia of speech severity score on the speech production factor. We recognize that repetition errors may not purely reflect speech production phenomena as greater difficulty with non-word versus real word tasks typically favors contribution from central language processes (see earlier discussion of this issue). However, the factor structure that emerged from the PCAs clearly distinguished a construct apart from semantics and phonological skill, and the repetition tasks loaded only on this speech production factor. Consistent with this interpretation, the factor was a strong predictor of performance on tasks with overt speech production (spoken naming and oral reading). There was small but significant weight of speech production on writing tasks, which may reflect a contributing role of subvocal speech during written spelling. In the broader context, the speech production factor was the strongest predictor of the WAB repetition composite score which includes single-word as well as sentence-level repetition. Long sentences and digit span tasks place additional demands on phonological working memory that should be distinct from speech production. This was evident from the more inclusive PCAs where digit span loaded on phonological skill more than speech production, regardless of whether the response was spoken or pointing. Thus, the speech production factor was most closely aligned with functions supported by the motor control network of the dorsal pathway. Mirman and colleagues similarly identified a speech production factor, which they distinguished from a “speech recognition” factor that had strong loadings from auditory minimal pair tasks (Mirman et al., 2015a; Thye and Mirman, 2018).

In relation to previous research, the “phonology” factor identified in most PCA studies to date appears to be best aligned with the “speech production” factor identified in our analyses. Lambon Ralph and colleagues referred to the factor with strong loadings from word and non-word repetition more broadly as “phonology,” in a manner that encompassed both central phonological processing along with the motor control network for speech (Lambon Ralph et al., 2002; Butler et al., 2014; Halai et al., 2017; Woollams et al., 2018). However, recognizing the limitations of word/non-word repetition to test phonology, Lambon Ralph et al. (2002) stated that the task “may not be the most sensitive measure of this type of deficit” (p. 63) as compared to tasks like “phonemic blending or segmentation.” Consistent with that view, they used non-word reading scores rather than spoken repetition as a proxy measure of phonology when predicting spoken naming ability. In our omnibus PCA, we similarly found reading of consonant-vowel-consonant non-words to load strongly on the phonological skill factor.

It is important to note that, although the phonological tasks in this study were weighted toward those that required sublexical processing of sounds and syllables, this should not be taken to mean that phonological support is only in the form of letter-sound transcoding. The phonology factor was a strong predictor of spoken naming, where responses were typically produced as lexical items, that is, with no evidence of a sounding out strategy. While somewhat difficult to explain, it seems that the sublexical manipulation tasks provide a measure of the status of the fully developed phonological sound system in literate individuals, and as such, the tasks are highly sensitive indicators of the integrity of central phonological representations supported by the dorsal pathway. Alternatively, it might be that sublexical phonological representations are automatically activated during naming and offer bottom-up feedback to aid phonological lexical selection (along with top–down semantic input), as in the interactive speech production model of Dell et al. (1997) and Schwartz et al. (2006). The latter explanation highlights the contribution of sublexical and lexical integration which has been the focus of some treatment protocols as discussed below.

Whereas we used “speech production” in reference to relatively peripheral processes, the term was used in several recent studies in relation to measures from spoken picture description, such as total number of spoken words and words per minute. These measures loaded on a separate factor that Halai et al. (2017) characterized as speech fluency or speech quanta and Ingram et al. (2020) referred to as speech production. It would appear that these constructs were intended to capture higher level, integrated aspects of spoken output on tasks that place considerable demands on central language components (e.g., semantics, phonology, syntax). That is quite different from teasing out peripheral aspects of speech production which are relevant to both word-level and narrative tasks. It will be important in future research to characterize the central and peripheral contributions to both spoken and written narrative production in addition to word-level language tasks.

Regarding written language processing, two unique factors consistently emerged from the PCA analyses: visual orthographic processing and allographic skill. The orthographic factor reflected strong loadings from the visual lexical decision tasks that simply require the determination of real words versus non-words. Given the anatomical integrity of orthographic processing regions (left VWFA) in our cohort, the functional impairment most likely reflects disruption of the bidirectional flow of information between phonology-orthography and semantics-orthography. Thus, we see that the orthographic processing system does not function properly without adequate input from the other two central language components. This is consistent with the impaired reading and spelling that is typically observed in those with left perisylvian damage and was illustrated by the regression models for reading and spelling for our cohort. Oral reading performance was strongly predicted by speech production, semantics, and phonological skill, with lesser weight from the visual processing component. As noted above, written spelling was strongly predicted by phonological skill with support from semantics and visual processing. Taken together, these findings demonstrate the interplay between the primary system for visual processing and other components of the language model. As expected, the visual orthographic factor did not contribute to non-visual tasks such as the spoken repetition or auditory comprehension composite scores from the WAB.

Allographic skill was captured by letter writing tasks including direct copying and case conversion tasks, that is, transcoding from lower to uppercase and vice versa. These tasks reflect peripheral aspects of handwriting including the ability to activate correct letter forms and the graphomotor skill to write them. Regression models affirmed that the allographic factor uniquely predicted performance on writing tasks and did not contribute to any spoken tasks. There was considerable variation in our cohort regarding the status of allographic skills. Given that left MCA can extend dorsally into superior parietal regions known to support visual-spatial skills necessary to generate appropriate letter shapes (Beeson P. et al., 2003; Purcell et al., 2011), it follows that some individuals may have allographic impairment while others do not.

In summary, the components that emerged from PCA reflect an integrated model of language that supports both spoken and written language processing. The shared components of semantics and phonological skill were strong predictors of word-level language performance, with phonology being particularly vulnerable to impairment in those with left perisylvian damage. Although critical orthographic processing regions were typically intact in our cohort, weakened phonological and semantic input to orthography disrupted reading and spelling abilities. Finally, spoken and written output were supported by peripheral components that engage the respective sensorimotor networks.

### Translation to clinical practice

The shared components model presented here highlights the common underlying support for spoken and written language, but there is often limited integration of spoken and written language profiles in clinical practice. The dataset for this study came from a heterogeneous group of individuals with respect to aphasia profiles and severity of language impairment. All the classic perisylvian aphasia types were represented (Broca’s, Conduction, Wernicke’s, Global) as well as a good number with evolved anomic aphasia and several who showed relatively well-recovered spoken language skills. In contrast to the diversity of aphasia types, the written language profiles documented in this cohort overwhelmingly reflected phonological or global agraphia accompanied by phonological or global alexia. Thus, the phonological impairment that was easily detectable on reading and spelling tasks was a unifying characteristic of those with left perisylvian damage. To this point, we are reminded of the paper by Patterson and Marcel (1992) who posed the question in relation to reading: is it Phonological ALEXIA or PHONOLOGICAL Alexia? Indeed, they demonstrated that it is PHONOLOGICAL Alexia, that is, an underlying impairment of phonology that is evident in reading non-words, and the same is true of PHONOLOGICAL agraphia (Henry et al., 2007; Rapcsak et al., 2009). In this study, we documented that phonological manipulation skills are also relevant to speech production tasks and are the most persistent deficit in those with left perisylvian/dorsal pathway damage, prompting us to consider whether we should refer to them as PHONOLOGICAL aphasias.

It is clear that an underlying phonological impairment is often marked and persistent following left perisylvian damage due to stroke (Patterson and Marcel, 1992; Henry et al., 2007; Rapcsak et al., 2009; Madden et al., 2018), as well as cortical atrophy to this region (Henry et al., 2012, 2016). As noted, this may be relatively obvious in written language, but the impact on spoken language may not be readily apparent. Spoken naming requires semantic activation of appropriate phonological codes, and lexical retrieval difficulties often reflect activation of conceptual knowledge, but difficulty bringing up the word: “I know what it is, I just can’t think of the name.” It appears that weakened phonology contributes to the access problem. If so, should phonological skill be a focus of behavioral treatment for both written and spoken language impairments? There are limited data to address this question, but several treatment studies have shown that phonological skills can improve in response to treatment and the outcomes include not only improved reading and spelling, but also improved naming ability in some participants (Beeson et al., 2010, 2018, 2019; Kendall et al., 2015; Johnson et al., 2019). In such cases, treatment was directed toward phonological awareness and manipulation skills in a manner that is distinct from training lexical retrieval strategies such as those that emphasize increased semantic activation (e.g., semantic feature analysis) or stimulation of specific phonological word forms (e.g., repetition priming). Thus, it appears that behavioral treatment to remediate this central phonological impairment has the potential to benefit spoken as well as written language. Again, the fact that sublexical phonological training improves phonological lexical selection seems to support the interactive model of Dell et al. (1997). That is, sublexical phonological information can boost and reinforce damaged/weakened lexical phonological representations in a bottom-up fashion allowing for lexical-sublexical integration to take place in addition to top-down semantic support.

In this study, we focused on word-level language skills; we did not address syntactic skills or the role of semantics and phonology at the sentence level. Of interest, however, are the findings from recent treatment research showing that strengthening phonological skills resulted in more correct information units in spoken narratives (Silkes et al., 2021) and improved grammatical/morphological structure of written sentences (Beeson et al., 2018, 2019). The latter is relevant to long-term recovery from aphasia, as written narratives are likely to show the effects of persistent phonological deficits after spoken language is relatively well recovered (DeMarco et al., 2017; Beeson et al., 2018).

Relevant to treatment outcomes, it is well established that individuals with weak non-verbal cognitive skills may have limited response to treatment (Helm-Estabrooks, 2002; Beeson P. M. et al., 2003; Lambon Ralph et al., 2010). In our cohort, many individuals showed relatively strong performance on the *Ravens Coloured Progressive Matrices*, but a subset was impaired in relation to controls. The PCA did not reveal a construct specific to non-verbal executive function/problem-solving ability that was detected in some studies (Butler et al., 2014; Tochadse et al., 2018; Ingram et al., 2020). Had we included other measures to sample this construct, a separate factor might have been detected. This issue warrants attention in future research as it may play a moderating role in response to treatment.

## Conclusion

The findings from this study support a shared components model of spoken and written language reflecting the interaction of semantics, phonology, and orthography. The derived factor scores for these central constructs along with measures of speech production and handwriting ability provided strong prediction of performance on word-level tasks. The characterization of phonological skill and distinction between central and peripheral processing components offers new insight in relation to previous research. These findings are relevant to neuropsychological models but also to research aiming to clarify the neural substrates of language. From a clinical perspective, the findings offer guidance regarding assessment tasks that are sensitive to core language components and the moderating effects of motor control networks and highlight the critical role of phonology in individuals with left perisylvian damage.

## Data availability statement

The raw data supporting the conclusions of this article will be made available by the authors, without undue reservation.

## Ethics statement

The studies involving human participants were reviewed and approved by Human Subjects Protection Program The University of Arizona. The patients/participants provided their written informed consent to participate in this study.

## Author contributions

PB contributed to the conceptualization and implementation of this study and took the lead on all behavioral data analyses and writing of the manuscript. KR took the lead in refinement of data collection protocols, managed and contributed to data collection throughout the study, coordinated participant recruitment, and contributed to the interpretation and writing of the manuscript. AS assisted with data management and reliability, contributed to lesion mapping, construction of lesion overlay figures, and manuscript editing. SR contributed to the conceptualization and design of the study, data interpretation, and writing of the manuscript. All authors contributed to the article and approved the submitted version.
