# Supplementary material for: Common predictors of spoken and written language performance in aphasia, alexia, and agraphia
Source: Front Hum Neurosci. 2022 Nov 7;16:1025468. doi: 10.3389/fnhum.2022.1025468 (PMC9677348; doi:10.3389/fnhum.2022.1025468)
Supplement: Supplementary file 1 [file Data_Sheet_1.docx]

Supplementary Tables

Table A. Results from Omnibus Principal Components Analysis Implemented Using All Test Scores (33) from 120 Participants (85 Language Impaired and 35 Neurotypical Controls). Factor scores greater than .50 in **bold**. Components: 1 = phonological skill, 2 = semantic processing, 3 = speech production, 4 = allographic skill, 5 = visual orthographic recognition.

|  | Component | 1 | 2 | 3 | 4 | 5 |
| --- | --- | --- | --- | --- | --- | --- |
|  |  | Phon | Semantic | Speech | Allog | Visual |
| 1 | Sound Replacement (spoken) | **0.90** | 0.19 | 0.17 | 0.14 | 0.17 |
| 2 | Spell Nonwords (write) | **0.90** | 0.20 | 0.18 | 0.15 | 0.18 |
| 3 | Read Nonwords (spoken) | **0.84** | 0.26 | 0.24 | 0.18 | 0.21 |
| 4 | Digit Span Forward (point) | **0.84** | 0.22 | 0.22 | 0.21 | 0.12 |
| 5 | Sound Deletion (spoken) | **0.80** | 0.36 | 0.26 | 0.15 | 0.15 |
| 6 | Sound Blending (spoken) | **0.80** | 0.28 | 0.24 | 0.16 | 0.08 |
| 7 | Spell Irregular Words (write) | **0.79** | 0.30 | 0.25 | 0.21 | 0.29 |
| 8 | Spell CVC Nonwords (write) | **0.78** | 0.40 | 0.27 | 0.18 | 0.17 |
| 9 | Spell Regular Words (write) | **0.78** | 0.31 | 0.28 | 0.21 | 0.26 |
| 10 | Digit Span Forward (spoken) | **0.78** | 0.07 | 0.39 | 0.09 | 0.11 |
| 11 | Sound Segmentation (written) | **0.70** | 0.44 | 0.31 | 0.27 | 0.16 |
| 12 | Transcode Letter-Sound (spoken) | **0.66** | 0.42 | 0.29 | 0.13 | -0.05 |
| 13 | Transcode Sound-Letter (write) | **0.65** | **0.51** | 0.32 | 0.23 | 0.06 |
| 14 | Read CVC Nonwords (spoken) | **0.64** | 0.44 | 0.47 | 0.15 | 0.17 |
| 15 | Boston Naming Test (spoken) | **0.63** | 0.47 | 0.45 | 0.03 | 0.22 |
| 16 | Sound Segmentation (spoken) | **0.61** | 0.41 | 0.42 | 0.14 | 0.07 |
| 17 | Written Word-Picture P48 (point) | 0.29 | **0.79** | 0.31 | 0.12 | 0.16 |
| 18 | AZ Semantic Test (point) | 0.32 | **0.76** | 0.08 | 0.28 | 0.13 |
| 19 | Spoken Word-Picture P47 (point) | 0.31 | **0.75** | 0.30 | -0.03 | 0.01 |
| 20 | Pyramids Palm Trees pics (point) | 0.27 | **0.72** | 0.08 | 0.33 | 0.21 |
| 21 | Synonym Judgment P49 (yes-no) | 0.43 | **0.67** | 0.28 | 0.00 | 0.24 |
| 22 | Repeat Words (spoken) | 0.28 | 0.22 | **0.86** | 0.05 | 0.10 |
| 23 | Repeat Nonwords (spoken) | 0.41 | 0.18 | **0.79** | 0.11 | 0.09 |
| 24 | Apraxia Rating (spoken)^1^ | 0.29 | 0.11 | **0.72** | -0.06 | -0.07 |
| 25 | Read Regular Words (spoken) | 0.48 | 0.46 | **0.61** | 0.08 | 0.25 |
| 26 | Read Irregular Words (spoken) | 0.51 | 0.45 | **0.60** | 0.06 | 0.26 |
| 27 | Upper-Lowercase Letter (write) | 0.32 | 0.36 | 0.03 | **0.77** | 0.07 |
| 28 | Direct copy words (write) | 0.06 | -0.13 | 0.05 | **0.77** | 0.07 |
| 29 | Ravens Coloured Prog M (point) | 0.20 | 0.16 | 0.05 | **0.72** | 0.16 |
| 30 | Lower-Uppercase Letter (write) | 0.27 | 0.40 | -0.12 | **0.71** | 0.11 |
| 31 | Visual Lex Decision P25 (mark) | 0.45 | 0.18 | 0.07 | 0.15 | **0.74** |
| 32 | Visual Lex Decision P27 (mark) | 0.33 | 0.31 | 0.02 | 0.19 | **0.73** |
| 33 | Visual Lex Decision P24 (mark) | 0.00 | 0.04 | 0.20 | 0.50 | **0.56** |
|  | Initial Eigenvalue | 19.88 | 2.72 | 1.73 | 1.31 | 1.09 |
|  | % Variance (rotated model) | 33.82 | 17.17 | 13.54 | 9.75 | 6.69 |
|  | Cumulative % Variance (rotated model) | 33.82 | 50.99 | 64.53 | 74.28 | 80.97 |

Extraction Method: Principal Component Analysis after Varimax Rotation with Kaiser Normalization.

Rotation converged in 6 iterations. Kaiser-Meyer-Olkin measure of sampling adequacy = .928.
Bartlett’s test of sphericity = 5732.11, *df* = 528, *p* <.001.

^1^Scale direction converted (lower = more impaired).

Table B. Results from Principal Components Analysis Implemented Using 26 Scores from 120 Participants (85 Language Impaired and 35 Neurotypical Controls). Factor scores greater than .50 in **bold**. 1 = phonological skills, 2 = semantic processing, 3 = allographic skill, 4 = speech production, 5 = visual orthographic recognition.

|  | Component | 1 | 2 | 3 | 4 | 5 |
| --- | --- | --- | --- | --- | --- | --- |
|  |  | Phon | Semantic | Allog | Speech | Visual |
| 1 | Sound Replacement (spoken) | **0.89** | 0.21 | 0.14 | 0.15 | 0.22 |
| 2 | Digit Span Forward (point) | **0.85** | 0.23 | 0.21 | 0.19 | 0.18 |
| 3 | Sound Blending (spoken) | **0.81** | 0.29 | 0.17 | 0.20 | 0.13 |
| 4 | Sound Deletion (spoken) | **0.80** | 0.37 | 0.16 | 0.22 | 0.19 |
| 5 | Digit Span Forward (spoken) | **0.80** | 0.09 | 0.10 | 0.35 | 0.15 |
| 6 | Spell CVC Nonwords (write) | **0.74** | 0.44 | 0.19 | 0.26 | 0.19 |
| 7 | Transcode Letter-Sound (spoken) | **0.67** | 0.43 | 0.13 | 0.27 | 0.00 |
| 8 | Sound Segmentation (written) | **0.67** | 0.48 | 0.27 | 0.28 | 0.18 |
| 9 | Sound Segmentation (spoken) | **0.64** | 0.42 | 0.13 | 0.39 | 0.13 |
| 10 | Read CVC Nonwords (spoken) | **0.63** | 0.47 | 0.16 | 0.43 | 0.18 |
| 11 | Transcode Sound-Letter (write) | **0.62** | **0.55** | 0.24 | 0.30 | 0.07 |
| 12 | Written Word-Picture P48 (point) | 0.28 | **0.80** | 0.12 | 0.27 | 0.17 |
| 13 | Spoken Word-Picture P47 (point) | 0.29 | **0.78** | -0.03 | 0.29 | 0.02 |
| 14 | AZ Semantic Test (point) | 0.31 | **0.76** | 0.27 | 0.05 | 0.16 |
| 15 | Pyramids Palm Trees pics (point) | 0.25 | **0.73** | 0.33 | 0.05 | 0.23 |
| 16 | Synonym Judgment P49 (yes-no) | 0.42 | **0.68** | -0.01 | 0.26 | 0.27 |
| 17 | Direct copy words (write) | 0.04 | -0.12 | **0.77** | 0.07 | 0.08 |
| 18 | Upper-Lowercase Letter (write) | 0.30 | 0.37 | **0.77** | 0.03 | 0.09 |
| 19 | Ravens Coloured Prog M (point) | 0.21 | 0.14 | **0.72** | 0.02 | 0.18 |
| 20 | Lower-Uppercase Letter (write) | 0.24 | 0.41 | **0.71** | -0.13 | 0.12 |
| 21 | Repeat Words (spoken) | 0.30 | 0.25 | 0.04 | **0.84** | 0.12 |
| 22 | Repeat Nonwords (spoken) | 0.43 | 0.20 | 0.10 | **0.77** | 0.12 |
| 23 | Apraxia Rating (spoken)^1^ | 0.30 | 0.14 | -0.07 | **0.73** | -0.04 |
| 24 | Visual Lex Decision P25 (mark) | 0.42 | 0.19 | 0.14 | 0.05 | **0.76** |
| 25 | Visual Lex Decision P27 (mark) | 0.31 | 0.31 | 0.18 | -0.01 | **0.75** |
| 26 | Visual Lex Decision P24 (mark) | -0.05 | 0.06 | 0.49 | 0.23 | **0.59** |
|  | Initial Eigenvalue | 14.31 | 2.59 | 1.49 | 1.17 | 1.05 |
|  | % Variance (rotated model) | 28.49 | 19.48 | 11.76 | 11.68 | 7.84 |
|  | Cumulative % Variance (rotated model) | 28.49 | 47.97 | 56.73 | 71.41 | 79.25 |

Extraction Method: Principal Component Analysis after Varimax Rotation with Kaiser Normalization.

Rotation converged in 6 iterations. Kaiser-Meyer-Olkin measure of sampling adequacy = .907.
Bartlett’s test of sphericity = 3537.50, *df* = 325, *p* <.001.

^1^Scale direction converted (lower = more impaired).

Table C. Results from Principal Components Analysis Implemented Using 14 Scores from all 123 Participants (87 Language Impaired and 36 Neurotypical Controls). Factor scores greater than .50 in **bold**. 1 = speech production, 2 = semantic processing, 3 = phonological skill, 4 = allographic skills, 5 = visual orthographic recognition.

|  | Component | 1 | 2 | 3 | 4 | 5 |
| --- | --- | --- | --- | --- | --- | --- |
|  |  | Speech | Semantic | Phon | Allog | Visual |
| 1 | Repeat Words (spoken) | **0.89** | 0.24 | 0.18 | 0.04 | 0.14 |
| 2 | Repeat Nonwords (spoken) | **0.82** | 0.21 | 0.32 | 0.08 | 0.16 |
| 3 | Apraxia Rating (spoken)^1^ | **0.81** | 0.11 | 0.18 | 0.00 | 0.00 |
| 4 | Written Word-Picture P48 (point) | 0.36 | **0.80** | 0.19 | 0.11 | 0.23 |
| 5 | Spoken Word-Picture P47 (point) | 0.34 | **0.80** | 0.24 | -0.03 | 0.07 |
| 6 | Pyramids Palm Trees pics (point) | 0.07 | **0.72** | 0.30 | 0.29 | 0.23 |
| 7 | Sound Replacement (spoken) | 0.27 | 0.21 | **0.85** | 0.14 | 0.29 |
| 8 | Sound Blending (spoken) | 0.32 | 0.26 | **0.82** | 0.17 | 0.19 |
| 9 | Sound Deletion (spoken) | 0.33 | 0.34 | **0.77** | 0.15 | 0.27 |
| 10 | Direct copy words (write) | 0.04 | -0.14 | 0.04 | **0.86** | 0.04 |
| 11 | Upper-Lowercase Letter (write) | 0.07 | 0.39 | 0.23 | **0.74** | 0.23 |
| 12 | Lower-Uppercase Letter (write) | -0.02 | 0.46 | 0.19 | **0.69** | 0.23 |
| 13 | Visual Lex Decision P25 (mark) | 0.16 | 0.12 | 0.29 | 0.14 | **0.86** |
| 14 | Visual Lex Decision P27 (mark) | 0.06 | 0.25 | 0.21 | 0.19 | **0.85** |
|  | Initial Eigenvalue | 6.95 | 1.97 | 1.07 | 1.02 | 0.75 |
|  | % Variance (rotated model) | 19.19 | 18.54 | 18.28 | 14.29 | 13.65 |
|  | Cumulative % Variance (rotated model) | 19.19 | 37.73 | 56.01 | 70.30 | 83.95 |

Extraction Method: Principal Component Analysis after Varimax Rotation with Kaiser Normalization.

Rotation converged in 6 iterations. Kaiser-Meyer-Olkin measure of sampling adequacy = .838.
Bartlett’s test of sphericity = 1414.59, *df* = 91, *p* <.001.

^1^Scale direction converted (lower = more impaired).
